# Bacterial and fungal community structures in Hulun Lake are regulated by both stochastic processes and environmental factors

**DOI:** 10.1128/spectrum.03245-23

**Published:** 2024-04-11

**Authors:** Yongquan Shang, Xibao Wang, Xiaoyang Wu, Huashan Dou, Qinguo Wei, Qi Wang, Gang Liu, Guolei Sun, Lidong Wang, Honghai Zhang

**Affiliations:** 1 School of Life Sciences, Qufu Normal University, Qufu, Shandong, China; 2 Hulunbuir Academy of Inland Lakes in Northern Cold & Arid Areas, Hulunbuir, China; Institut Ruder Boskovic, Zagreb, Croatia

**Keywords:** bacterial community, fungal community, sediment, water, community assembly, Hulun Lake

## Abstract

Microorganisms are a crucial component of lake ecosystems and significant contributors to biogeochemical cycles. However, the understanding of how primary microorganism groups (e.g., bacteria and fungi) are distributed and constructed within different lake habitats is lacking. We investigated the bacterial and fungal communities of Hulun Lake using high-throughput sequencing techniques targeting 16S rRNA and Internal Transcribed Spacer 2 genes, including a range of ecological and statistical methodologies. Our findings reveal that environmental factors have high spatial and temporal variability. The composition and community structures vary significantly depending on differences in habitats. Variance partitioning analysis showed that environmental and geographical factors accounted for <20% of the community variation. Canonical correlation analysis showed that among the environmental factors, temperature, pH, and dissolved oxygen had strong control over microbial communities. However, the microbial communities (bacterial and fungal) were primarily controlled by the dispersal limitations of stochastic processes. This study offers fresh perspectives regarding the maintenance mechanism of bacterial and fungal biodiversity in lake ecosystems, especially regarding the responses of microbial communities under identical environmental stress.

**IMPORTANCE:**

Lake ecosystems are an important part of the freshwater ecosystem. Lake microorganisms play an important role in material circulation and energy flow owing to their unique enzymatic and metabolic capacity. In this study, we observed that bacterial and fungal communities varied widely in the water and sediments of Hulun Lake. The primary factor affecting their formation was identified as dispersal limitation during stochastic processes. Environmental and geographical factors accounted for <20% of the variation in bacterial and fungal communities, with pH, temperature, and dissolved oxygen being important environmental factors. Our findings provide new insights into the responses of bacteria and fungi to the environment, shed light on the ecological processes of community building, and deepen our understanding of lake ecosystems. The results of this study provide a reference for lake management and conservation, particularly with respect to monitoring and understanding microbial communities in response to environmental changes.

## INTRODUCTION

The Earth’s hydrosphere includes lakes, which are vital sources of freshwater (1, 2). Microbial communities are crucial components of lake ecosystems (3, 4), which act as both producers and decomposers of organic matter and regulate chemical cycles and other biological components within these ecosystems (5–9). Consequently, studying the characteristics of microbial communities in lakes is of utmost biological importance.

The crucial biological role played by microbial communities within lake ecosystems has spurred notable advancements in research. The characteristics of lake-dwelling bacteria are strongly associated with the lake environment (10–12). There are significant environmental differences between lake sediments and the overlying water column, enhancing the diversity in the structural composition of bacteria (13). Moreover, bacterial diversity is influenced by the nutrient status and seasonal changes of the lake (14). Salinity is another factor that can considerably influence the structural composition of microbial communities within lake ecosystems (15). The structure of bacterial communities within lake sediments can vary significantly at different depths (16). The characteristics of lake bacterial communities are influenced by the lake water environment (17). The composition of bacterial communities within lake ecosystems is closely associated with various ecological and environmental factors (18, 19). The breakdown of organic matter and the cycling of nutrients are driven by microorganisms (7, 20). A clear functional overlap exists between resource utilization by bacteria and fungi (21). Evidence also suggests that the interactions between bacteria and fungi affect various ecosystem processes (22). Nevertheless, the majority of lake studies have primarily focused on bacterial communities, with a relatively lesser exploration of fungal communities. Therefore, there is a need for an in-depth exploration and concurrent investigation of both bacterial and fungal communities.

To understand the mechanisms influencing microbial communities in lakes, it is crucial to investigate the factors driving community stability (23, 24). Many deterministic factors, such as pH and organic matter content, affect lake microbial communities (25, 26). Additionally, microorganisms are subject to stochastic processes (27). In certain undisturbed environments (e.g., permafrost soils or groundwater), stochastic processes play a central role in prokaryotes (28, 29). Microbial community assemblages within estuarine wetland soils are regulated by distinct stochastic processes (30). However, there is insufficient research on the influence of both deterministic and stochastic factors on microbial communities.

Hulun Lake is located within a semi-arid, high-latitude grassland region of Inner Mongolia, northern China. It is an important ecological barrier, playing a vital role in regional ecological protection (31). Microorganisms in the lake undergo changes due to seasonal and environmental factors (32). However, whether lake bacteria and fungi are responding to environmental factors and community assemblage remains to be explored.

In this study, we aimed to observe the structure of microbial communities (bacteria and fungi) in Hulun Lake under varying environmental conditions and explore the mechanisms driving community formation. Specifically, we sought to answer three questions: (i) Is there a difference in the structure of bacteria and fungi in different habitats within the lake? (ii) Are lake bacteria and fungi driven by similar environmental factors? (iii) What are the underlying mechanisms governing the community assemblage of microorganisms in the lake?

## MATERIALS AND METHODS

### Study area

Hulun Lake (48°30′40″–49°20′40″N, 117°00′10″–117°41′40″E) is located next to Manzhouli City. It is part of the Erguna River System, fed by three rivers, the Urxun, Krulun, and Xinkai (Hulun Lake chronicle). The lake’s geographical and underlying surface environments result in a large temperature variation throughout the year, with an annual average temperature of 0.3°C (32).

### Sample collection

To determine differences between microbial communities (bacteria and fungi) in different seasons, we collected 38 samples from 22 sites in the summer and 32 samples from 21 sites in the winter of 2018 (Fig. S1). Samples were collected from each sampling point, 0.5 m below the lake water surface using a 2.5 L organic glass water sampler. Prior to use, collection devices were cleaned and rinsed with alcohol and ultrapure water, respectively. Prior to sampling at each sampling point, the collection devices were first rinsed with lake water. The samples were placed in ice boxes for refrigeration immediately after collection until the time they were transported to the laboratory. Collected samples were partitioned into three portions. The first and second portions were filtered using 0.22 and 0.45 µm microporous membranes, respectively. The filtered membrane was stored at −80°C. Environmental factors for all the samples were determined immediately using previously described methods (32). A Petersen mud sampler (CN-100, 5L) was used for surface sediment sample collection, and the samples were stored at −80°C. The sample groups are presented in Table 1.

**TABLE 1:** Bacterial and fungal sample grouping in summer and winter

Sample type	Summer	Winter	Combined analysis grouping^*a*^
Bacterial water samples	HL group	WHL group	H-WH
Bacterial sediment samples	NS group	WHLN group	N-WN
Fungal water samples	TH group	TWHL group	TH-TWH
Fungal sediment samples	TNS group	TWHLN group	TN-TWN

^*a*^ Grouping of combined bacterial and fungal analyses.

### DNA extraction and sequencing

HiPure Soil DNA Kit and HiPure Stool DNA Kit (Magen, China) were used for DNA extraction. We amplified the V3–V4 regions of bacterial 16S rRNA genes (primers: 341F and 806R) and Internal Transcribed Spacer (ITS)2 rRNA region of fungi (primers: ITS3_KYO2 and ITS4). Previously described methods were used to conduct PCR amplification (33, 34). Sequencing was performed using Illumina HiSeq2500 (Guangzhou Genedenovo Biotechnology Co., Ltd.).

### Sequenced data processing

To ensure data quality, we removed adaptor and primer sequences and low-quality reads using FASTP (https://github.com/OpenGene/fastp) (35). FLASH (version 1.2.11) was used to merge clean paired-end reads (36). QIIME (version 1.9.1) was used to filter noise sequences (37). UCHIME was used to identify chimeric sequences. The high-quality amplicons were clustered into operational taxonomic units (OTUs) using UPARSE (version 9.2.64) with tags of ≥97% similarity. The SILVA and UNITE databases were used to annotate 16S rRNA and ITS sequences, respectively (38). Rarefaction curves were generated using the Origin software to evaluate the sequencing depth of the data (Fig. S2).

### Diversity analyses

Various diversity indices were calculated using QIIME (v1.9.1). The alpha diversity index was tested for differences using Welch’s *t*-test. GBMPlus was used to evaluate the relative effects of alpha diversity on environmental factors. The QIIME software was used to generate the Bray–Curtis distance matrices. Non-metric multidimensional scaling (NMDS) was generated using R with the vegan package (version 2.5–6) and plotted using the ggplot2 package. A statistical test of the ratio difference between groups was conducted using R with the vegan package. The psych package in R was used to perform a Pearson correlation analysis of the diversity index. The vegan package was used to conduct PERMANOVA in R. Bray–Curtis dissimilarities were used to detect significant correlations using the Procrustes test (999 times, bacterial and fungal).

### Statistical analyses

Using the vegan package, we performed canonical correlation analysis (CCA) to examine the relationships between the microbes and the environment. Additionally, variance partitioning analysis (VPA) was performed using Biozeron Cloud Platform. To explore co-occurrence patterns between microbial taxa, we used the top 100 abundant OTUs to construct networks and applied a Spearman correlation coefficient (*r*) threshold of >0.6 with *P*-values < 0.05. Gephi 0.9.1 was used for network visualization (39). To study ecological community assemblages, we calculated the beta nearest taxon index using R (40).

## RESULTS

### Environmental factors

The environmental factors included in this study are listed in Table S1. Temperature showed seasonal variation (−1.3 to 29.5°C). The pH ranged from 7.1 to 9.1, indicating that the water of Hulun Lake was weakly alkaline. Total nitrogen (TN) ranged from 2.02 ± 1.65 mg/L (winter) to 2.03 ± 1.05 mg/L (summer). Total phosphorus (TP) ranged from 0.14 ± 0.19 mg/L (winter) to 0.21 ± 0.08 mg/L (summer). Specifically, a large variation in the NH_4_^+^-N content was observed between summer and winter, ranging from 0.12 ± 0.06 mg/L (winter) to 2.98 ± 9.69 mg/L (summer). The values for NH_4_^+^-N content also varied across different sampling sites. Moreover, we found that temperature had a markedly negative effect on pH, electrical conductivity (EC), and chemical oxygen demand (COD), whereas pH had a markedly positive effect on EC, P, and COD. In summer, EC had a significant positive effect on TP, TN, and COD (*P* < 0.05; Fig. S3A). In winter, pH had a significant positive effect on dissolved oxygen (DO), and EC had a significant positive effect on N and COD (*P* < 0.05; Fig. S3B).

### Microbial α-diversity

From the 70 samples of the bacterial community, a total of 4,692,517 effective tags were obtained. Through cluster analysis with 97% similarity, 11,650 OTUs were generated, of which the number and percentage of unknown OTUs at different levels of classification were as follows: phylum (486, 4.1%), class (2,499, 21.4%), order (4,718, 40.4%), family (5,897, 50.6%), and genus (8,525, 73.1%) (Table S2). According to Shannon’s rarefaction curves, when the sequence coverage reached 4000, the curve tended to flatten (Fig. S2A). Therefore, most bacteria in this study were covered by the sequencing reads. The mean values of the ACE, Shannon, and Simpson indices were 3787.69, 7.07, and 0.95, respectively (Table S3).

Using ITS amplification sequencing, 27,077 fungi effective tags were obtained from 70 samples. Based on cluster analysis, 968 OTUs with 97% similarity were generated, of which the number and percentage of unknown OTUs at different levels of classification were as follows: phylum (391, 40.3%), class (435, 44.9%), order (463, 47.8%), family (541, 55.8%), and genus (620, 64%) (Table S4). According to Shannon’s rarefaction curves, when the sequence coverage reached 2000, the curve tended to flatten, and Good’s coverage was >92% (Fig. S2B). Thus, the sequencing depth in this study covered most fungal communities, thereby meeting the requirements of subsequent analyses.

**Fig 1:**
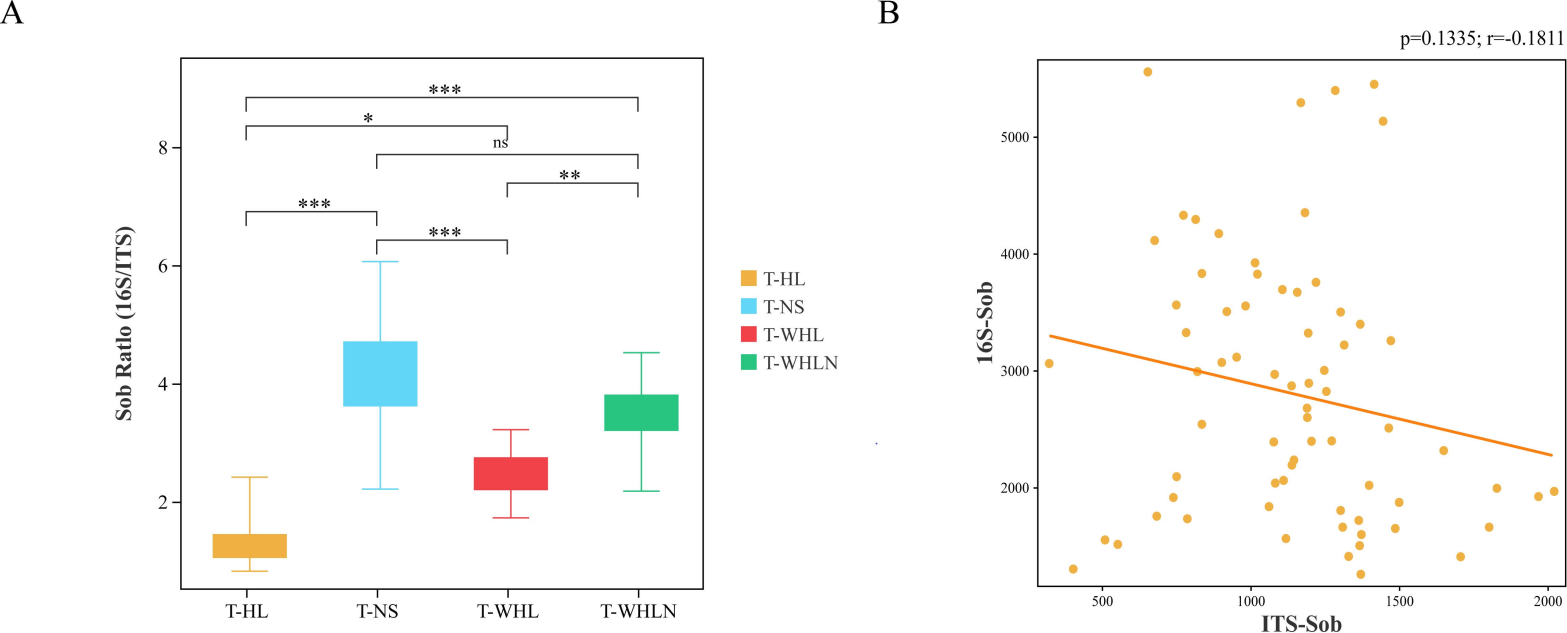
(**A**) Pairwise comparisons of Bray–Curtis dissimilarity values between different groups for 16S rRNA gene and ITS2 sequencing data (*P < 0.05, **P < 0.01, ***P < 0.001). (**B**) Correlation analysis between 16S and ITS was conducted based on the Mantel test, where *r* represents correlation strength and *P*-value represents correlation significance. ITS, internal transcribed spacer; T-HL, bacterial and fungal water samples in summer; T-NS, bacterial and fungal sediment samples in summer; T-WHL, bacterial and fungal water samples in winter; T-WHLN, bacterial and fungal sediments samples in winter.

The Simpson indices in Hulun Lake during the summer and winter seasons showed significant differences only in fungal sediment samples (Welch’s *t*-test, *P* < 0.05; Table 2). We explored the equilibrium between bacterial and fungal diversity in Hulun Lake. The fungal-to-bacterial SOB ratio was also determined. No statistically significant differences were observed between the T-WHLN and T-NS groups. Nevertheless, there were significant differences among the other groups (Fig. 1A). Pearson correlation analysis revealed no significant correlation between bacterial and fungal alpha diversity values (Fig. 1B).

**TABLE 2:** Seasonal differences in bacterial and fungal sample group Simpson index values using Welch’s *t*-test^*a*^

Sample type	Group	*P*-value
Bacterial	HL vs WHL	0.2749
	NS vs WHLN	0.0560
Fungal	TH vs TWHL	0.6654
	TNS vs TWHLN	0.0346

^*a*^ HL, bacterial water samples in summer; NS, bacterial sediment samples in summer; WHL, bacterial water samples in winter; WHLN, bacterial sediment samples in winter; TH, fungal water samples in summer; TNS, fungal sediment samples in summer; TWHL, fungal water samples in summer in winter; TWHLN, fungal sediment samples in winter.

### Correlations of bacteria and fungi α-diversity with environmental factors

**Fig 2:**
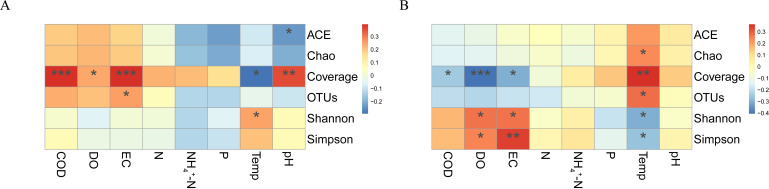
Correlation analysis between α-diversity indices and environmental factors for (**A**) bacteria and (**B**) fungi (*P < 0.05, **P < 0.01, ***P < 0.001).

In the bacterial community (Fig. 2A), statistically significant positive correlations with coverage were found for COD (*R* = 0.39, *P* < 0.001), DO (*R* = 0.24, *P* < 0.05), EC (*R* = 0.39, *P* < 0.001), and pH (*R* = 0.36, *P* < 0.01). Temperature was significantly negatively correlated with coverage (*R* = −0.29, *P* < 0.05), and pH was significantly negatively correlated with ACE (*R* = −0.24, *P* < 0.05). In the fungal community (Fig. 2B), statistically significant negative correlations with coverage were found for COD (*R* = −0.25, *P* < 0.05), DO (*R* = −0.41, *P* < 0.001), and EC (*R* = −0.30, *P* < 0.05).

### Microbial community composition

**Fig 3:**
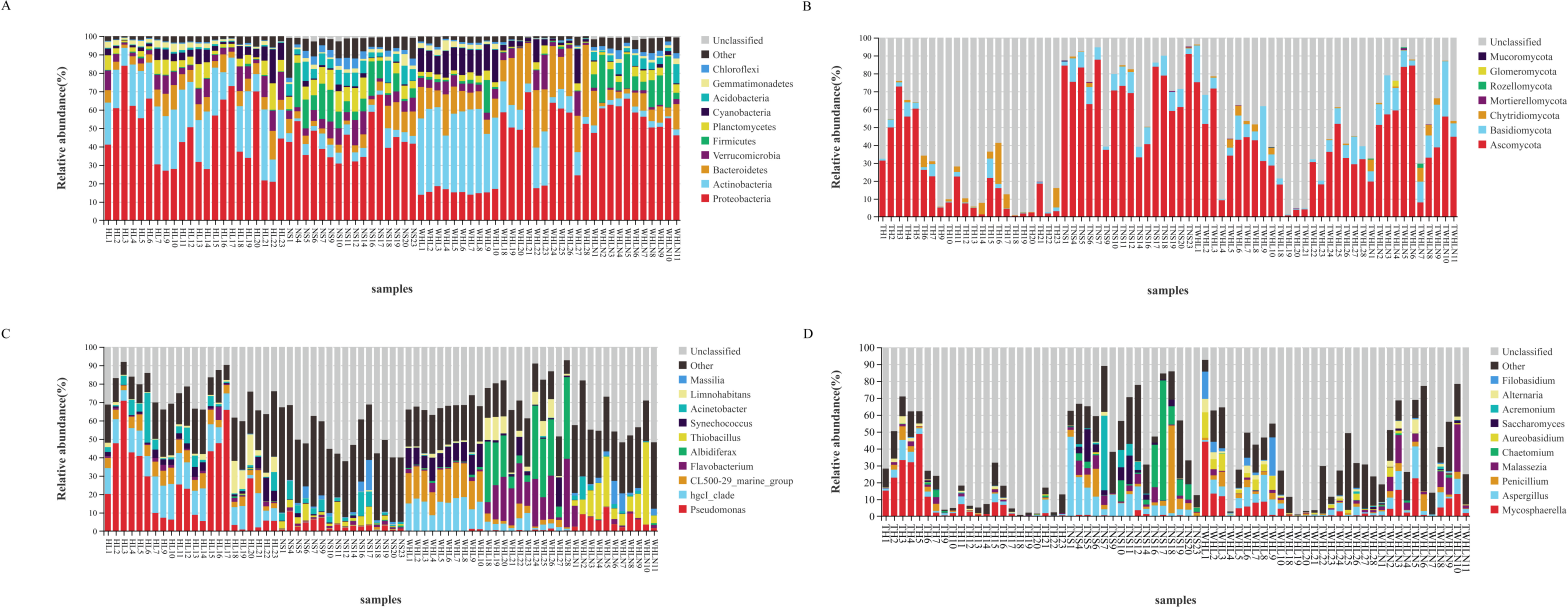
Microbial community composition of water and sediments in Hulun Lake. Relative abundances of dominant (**A**) bacterial and (**B**) fungal phyla; relative abundances of dominant bacterial (**C**) and (**D**) fungal genera.

The bacterial community comprised 56 phyla, 153 classes, 209 orders, and 735 genera. Proteobacteria was the dominant phylum, accounting for 44.18%, followed by Actinobacteria (14.41%), Bacteroidetes (9.89%), Verrucomicrobia (4.99%), Firmicutes (6.05%), Planctomycetes (4.78%), Cyanobacteria (3.82%), Acidobacteria (3.03%), Gemmatimonadetes (1.72%), Chloroflexi (1.81%), and Parcubacteria (1.08%) (Fig. 3A). At the genus level (Fig. 3C), *Pseudomonas* (8.54%) had the highest relative abundance, followed by *hgcI_clade* (5.48%), *CL500-29_marine_group* (3.99%), *Thiobacillus* (3.98%), *Flavobacterium* (3.52%), *Albidiferax* (3.04%), *Acinetobacter* (2.76%), *Synechococcus* (2.45%), *Limnohabitans* (1.81%), *Massilia* (1.43%), and *Tumebacillus* (1.40%).

The fungal community comprised 7 phyla, 25 classes, 64 orders, and 203 genera, with Ascomycota being the most dominant phylum (44.05%; Fig. 3B). Basidiomycota and Chytridiomycota had relative abundances of 5.08% and 1.82%, respectively. At the genus level (Fig. 3D), *Aspergillus* (5.83%) had the highest relative abundance, followed by *Mycosphaerella* (5.43%), *Malassezia* (3.21%), *Penicillium* (3.07%), *Chaetomium* (2.63%), *Saccharomyces* (1.21%), *Acremonium* (1.18%), *Alternaria* (1.06%), and *Aureobasidium* (1.03%).

### Microbial β-diversity

**Fig 4:**
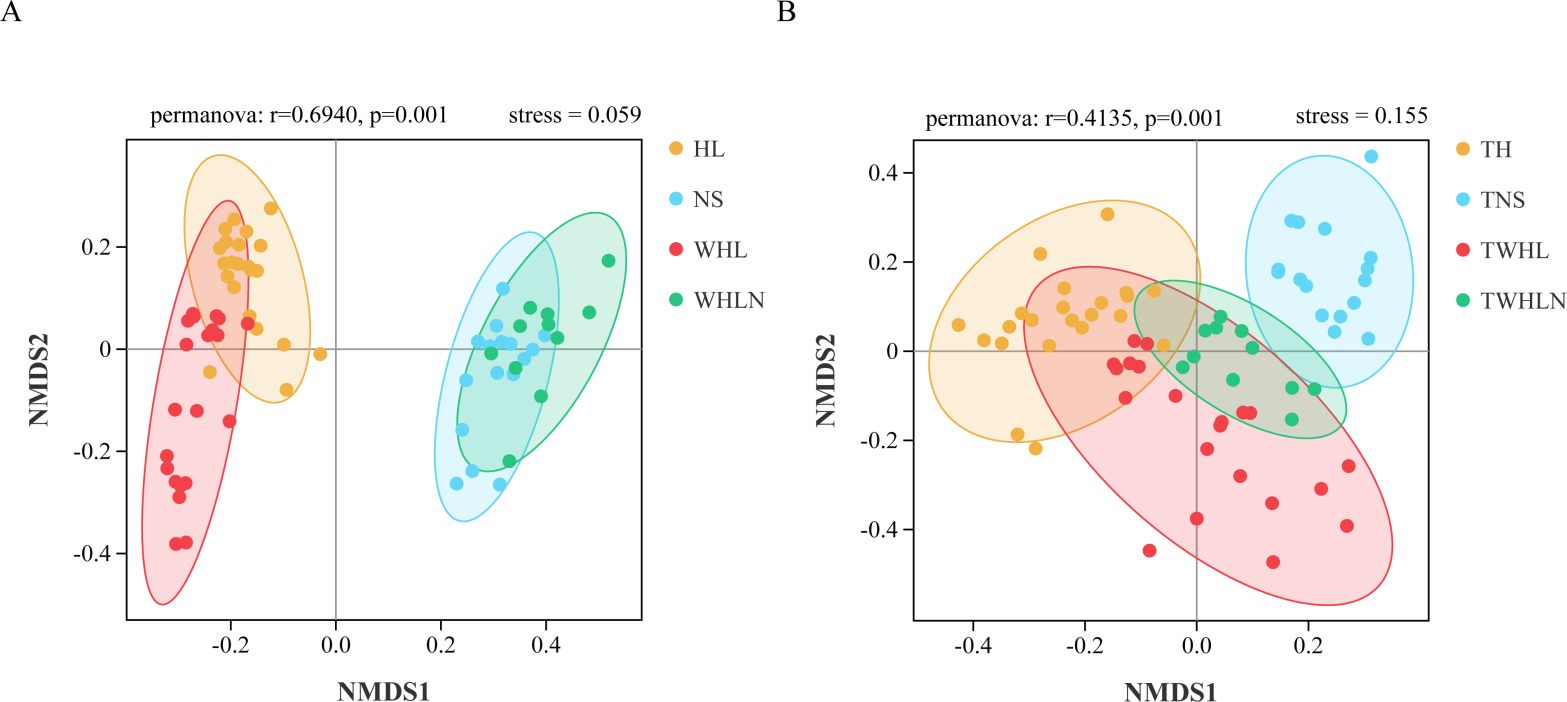
Non-metric multidimensional scaling (NMDS) of (**A**) bacteria and (**B**) fungal communities based on Bray–Curtis distances (permanova: *P* = 0.001). HL, bacterial water samples in summer; NS, bacterial sediment samples in summer; WHL, bacterial water samples in winter; WHLN, bacterial sediment samples in winter; TH, fungal water samples in summer; TNS, fungal sediment samples in summer; TWHL, fungal water samples in summer in winter; TWHLN, fungal sediment samples in winter.

To compare the differences in the community structure, we used the NMDS of the Bray–Curtis distance analysis (Fig. 4). The bacterial community had a stress value of 0.059, with clear separation samples in different groups on two axes (Fig. 4A, PERMANOVA: *P* = 0.001). However, seasonal distinctions were not evident in sediments. The fungal community had a stress value of 0.155, with obvious clustering among the samples, but no clear differences between sample types were noted (Fig. 4B, PERMANOVA: *P* = 0.001). This indicates that the fungal communities have similar community characteristics and that seasonal differences are not significant. In summary, both bacterial and fungal community distinctions between habitat types are more pronounced than seasonal distinctions.

**Fig 5:**
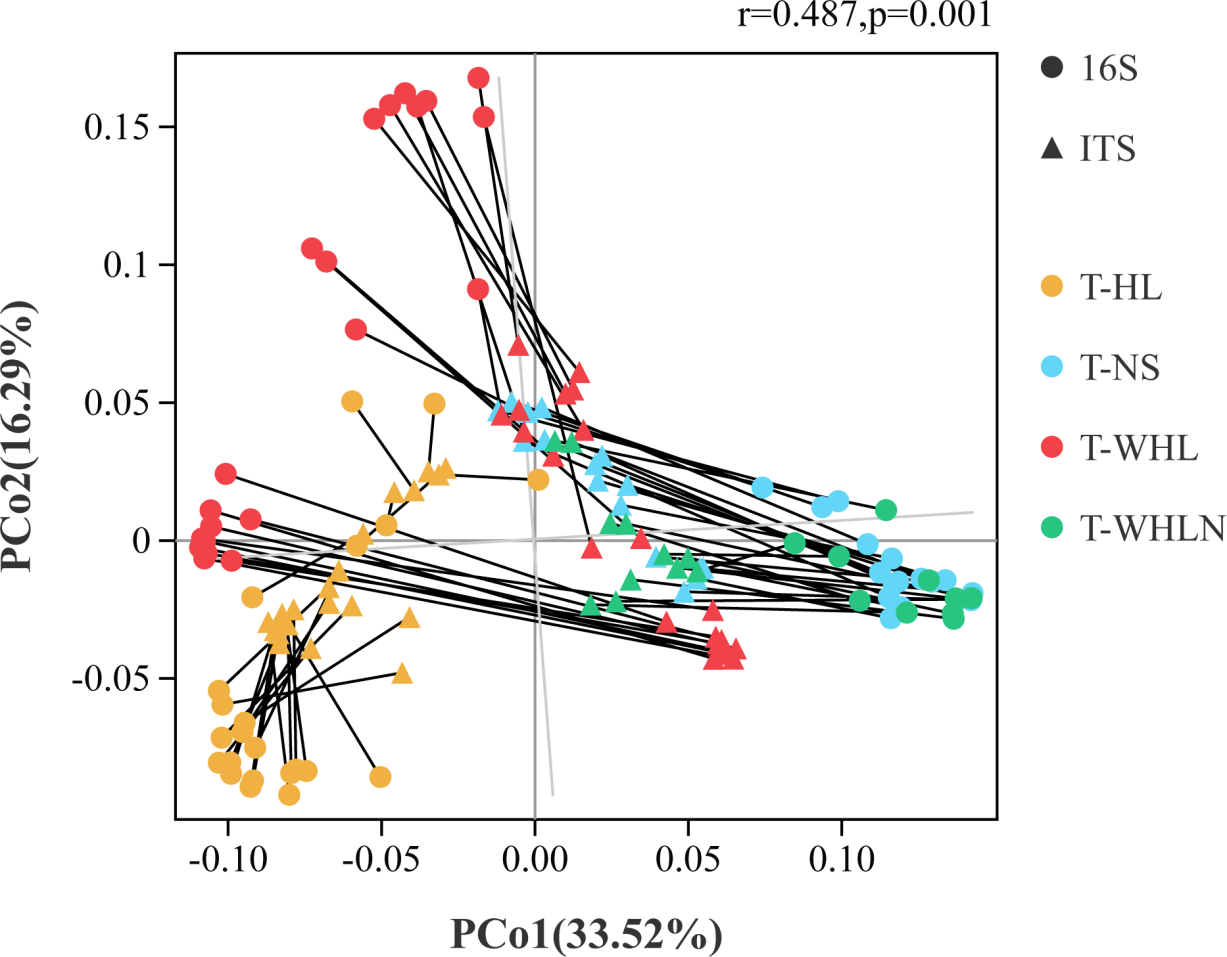
Procrustes test depicting the significant correlation between bacterial and fungal communities based on Bray−Curtis dissimilarity metrics (sum of squares *M*^2^ = 0.7626, *r* = 0.487, *P* = 0.001, 999 permutations). T-HL, bacterial and fungal water samples in summer; T-NS, bacterial and fungal sediment samples in summer; T-WHL, bacterial and fungal water samples in winter; T-WHLN, bacterial and fungal sediment samples in winter.

To assess whether bacteria and fungi responded equally to grouping differences, we performed the Procrustes test. At the level of OTUs, this test revealed a significant correlation between bacteria and fungi (*M*^2^ =  0.7626, *R*  =  0.487, *P*  =  0.001, permutations  =  999; Fig. 5).

### Relationships between microbial communities and environmental factors

**Fig 6:**
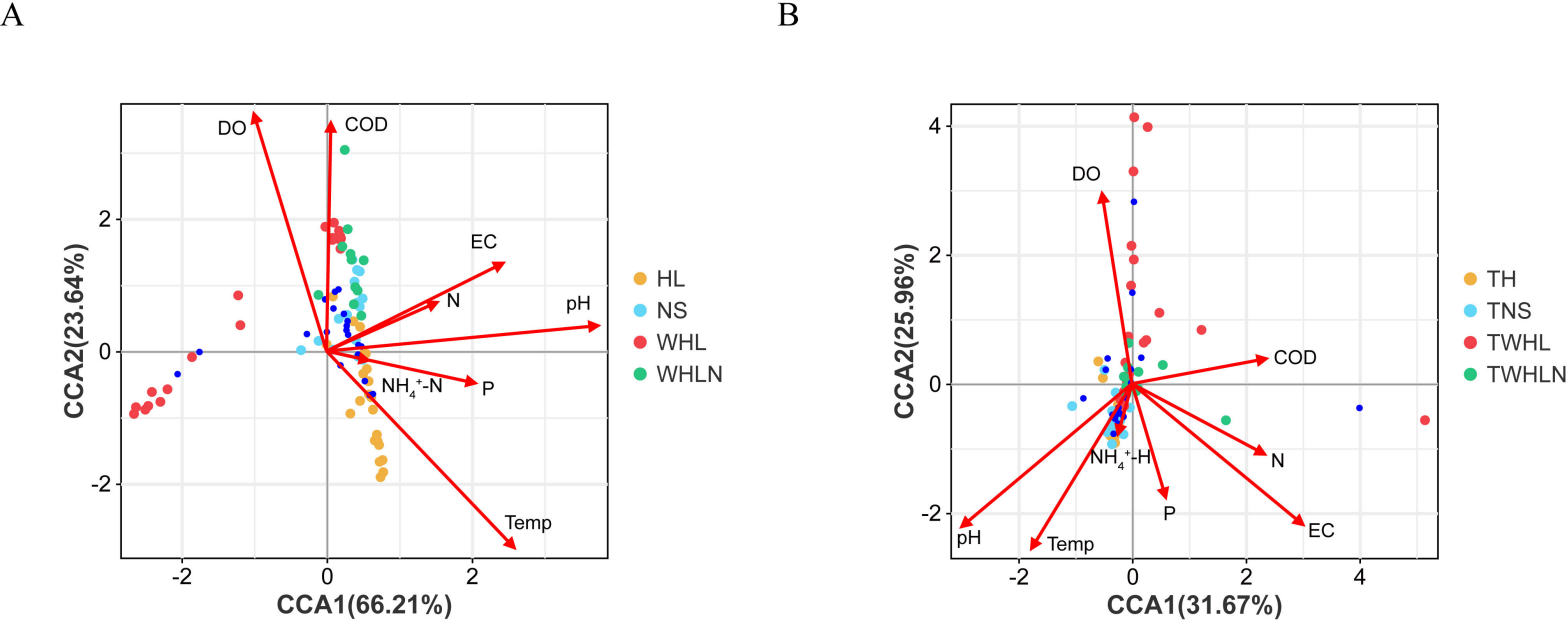
Canonical correspondence analysis based on (**A**) bacterial communities and (**B**) fungal communities with respect to OTU level and environmental factors (red arrows). The top 20 most abundantly classified bacterial and fungal OTUs (97% sequence similarity) in the samples. OTU, operational taxonomic unit; HL, bacterial water samples in summer; NS, bacterial sediment samples in summer; WHL, bacterial water samples in winter; WHLN, bacterial sediment samples in winter; TH, fungal water samples in summer; TNS, fungal sediment samples in summer; TWHL, fungal water samples in summer in winter; TWHLN, fungal sediment samples in winter.

Environmental and geographical factors were analyzed to determine their influence on microbial communities. VPA revealed that environmental factors had a greater influence on bacterial and fungal communities (14.8% and 12.4%, respectively) than did geographical factors (3.3% and 3.3%, respectively) (Fig. S4). However, environmental and geographical factors could not explain approximately 80% of the variation in these communities (bacteria: 81.2%, fungi: 83.3%; Fig. S4). CCA accounted for 89.85% of the explanation for the variation in the bacterial community structure in the first two axes (Fig. 6A). The bacterial community structure was most influenced by the pH (*R*^2^ = 0.77), temperature (*R*^2^ = 0.72), DO (*R*^2^ = 0.53), and COD (*R*^2^ = 0.43). The fungal community structure was most influenced by EC (*R*^2^ = 0.75), pH (*R*^2^ = 0.73), temperature (*R*^2^ = 0.49), and DO (*R*^2^ = 0.45) (Fig. 6B).

### Community assemblages of bacteria and fungi

**Fig 7:**
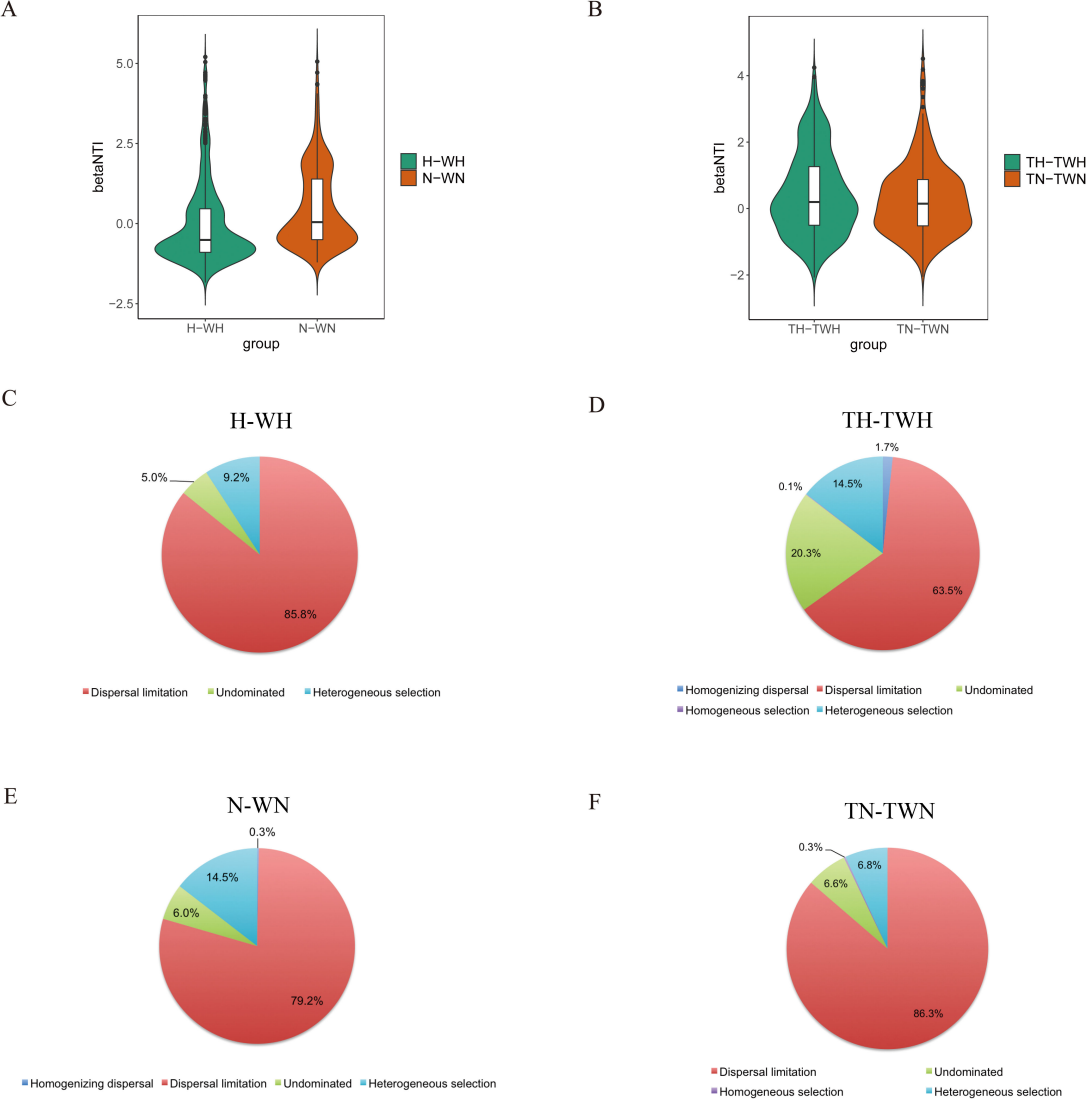
Distribution of beta nearest taxon index in the (**A**) bacterial and (**B**) fungal communities. Community assemblage mechanisms in the bacterial and fungal communities for the (**C**) H-WH, (**D**) TH-TWH, (**E**) N-WN, and (**F**) TN-TWN groups. H-WH, bacterial water samples in summer and winter; TH-TWH, fungal water samples in summer and winter; N-WN, bacterial sediment samples in summer and winter; TN-TWN, fungal sediment samples in summer and winter.

Since environmental and geographical factors did not account for the majority of the microbial community variation, we separately analyzed the community assemblages of different groups of bacteria and fungi. For the microbial communities, the values of NTI for the majority of the samples were between −2 and 2, indicating that community assembly was a stochastic process (Fig. 7A and B). For the bacterial community, dispersal limitation was the dominant driver of community assemblage in water (85.8%) and sediments (79.2%) of Hulun Lake (Fig. 7C and E). Dispersal limitation was also the dominant driver for the fungal community (water: 63.5%, sediments: 86.3%, Fig. 7D and F). Thus, dispersal limitation was the dominant driver of community assemblage.

To better understand the stochastic process, we explored the correlation between environmental factors and NTI using the Mantel text. The results indicate that environmental variables affect fungal communities much more than they do bacterial communities (Table S5).

### Microbial network analysis

**Fig 8:**
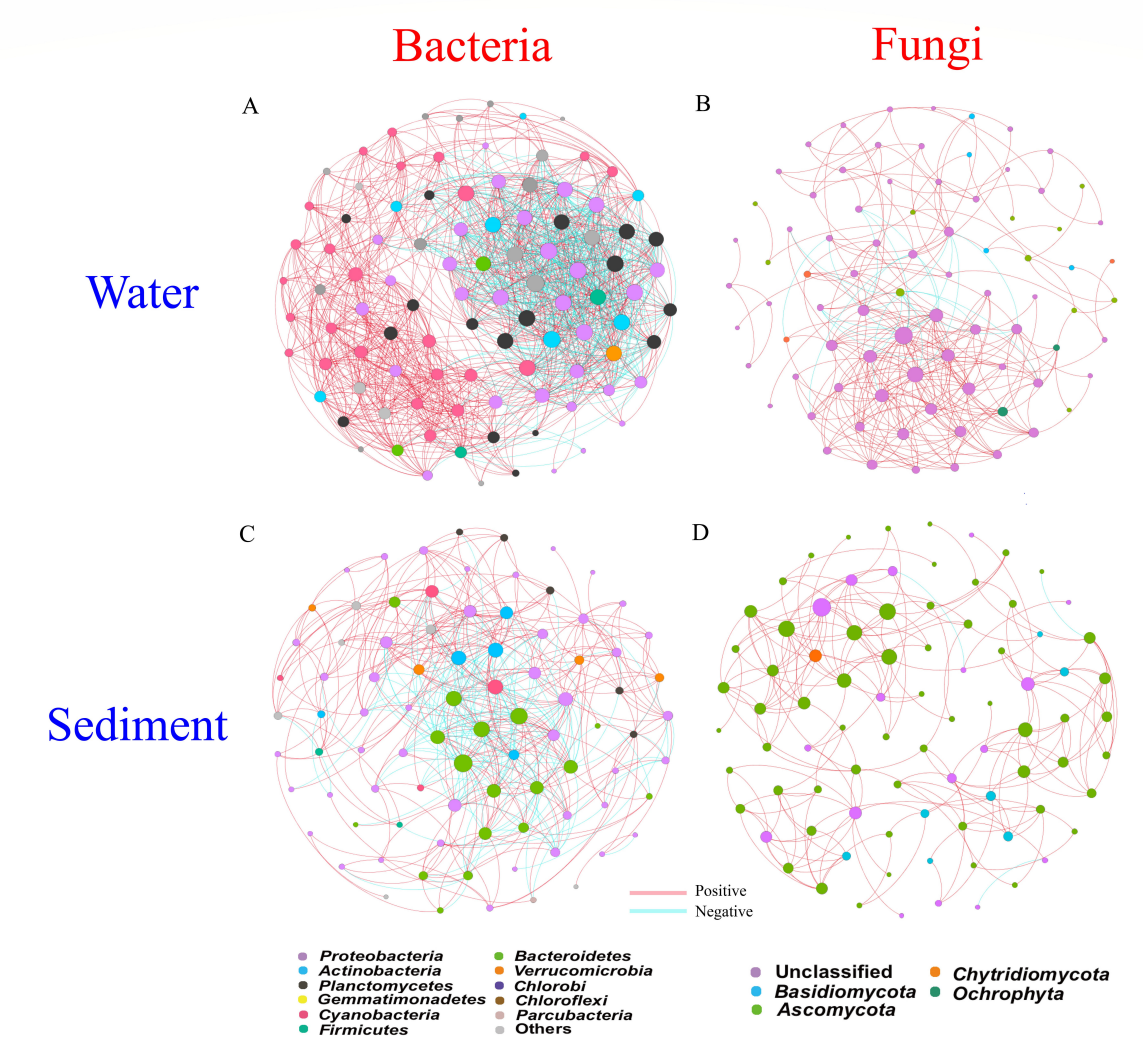
Microbial correlation networks in the (**A**) H-WH, (**B**)TH-TWH, (**C**) N-WN, and (**D**) TN-TWN groups. Different colored nodes represent different phyla. Node size represents the OTU degree. OTU, operational taxonomic unit; H-WH, bacterial water samples in summer and winter; TH-TWH, fungal water samples in summer and winter; N-WN, bacterial sediment samples in summer and winter; TN-TWN, fungal sediment samples in summer and winter.

We examined potential interactions between bacterial and fungal taxa using co-occurrence networks. In the network, most nodes had high connections (Fig. 8). We observed more edges, a larger average degree and graph density, and a higher average clustering coefficient for bacteria (Table S6).

## DISCUSSION

The microbial community composition of Hulun Lake has been widely reported; however, these reports focused on specific microbial taxa (41). In this study, the microbial communities (including bacterial and fungal) of the Hulun Lake basin were investigated. The two communities were evaluated based on their relative responses to environmental factors and geographic locations. Community assemblages were analyzed using the null model.

Each physicochemical index at different sampling sites in Hulun Lake showed large differences when the data for summer and winter were compared. Certain indices also showed some variability in the same season, thereby indicating a high spatial and temporal variability of the water environment. In the Hulun Lake basin, the N and P contents were higher than those in natural rivers and lakes, indicating a certain degree of eutrophication (42). We speculate that this may be related to the continuous reduction of water volume in recent years, pollutants in cattle and sheep manure washed with rainwater, and other factors (43).

Although the Simpson index takes into account both species richness and evenness, it is more sensitive to dominant species and focuses on the most abundant species while being less sensitive to species richness (44, 45). In this study, the Simpson index difference test revealed significant differences only in the sediment of fungi. There were similar dominant species of bacteria and fungi in the water samples. We cannot rule out that the results of the Simpson index reflect this. In partial agreement with previous studies (40), there were no significant differences in species richness between the T-WHLN and T-NS groups, whereas significant differences were observed among all other subgroups. This may be associated with sediments and water having different habitats (46). Owing to wind and temperature, the water of Hulun Lake constantly changes, impacting the microbial community. The sediment environment is more complex but relatively stable compared with the water column; therefore, bacteria and fungi living in the sediment did not show a difference in abundance. Additionally, we found that the diversity responded differently to grouping differences, which is consistent with previous findings (30).

Similar to the results of a study on Chaohu Lake (43), Proteobacteria, Actinobacteria, and Bacteroidetes were the three most abundant phyla in this study (47). Some studies suggest that these three phyla are associated with the metabolism of nutrients in water, which may be related to some degree of eutrophication in these two lakes (48, 49). At the genus level, *Pseudomonas*, *hgcI_clade,* and *CL500_29_marine_group* were the most abundant genera. All these genera are related to the metabolism of pollutants and purification of water (50, 51). Hulun Lake had poor water quality during sampling, which may have contributed to this result. Previous studies have also found that Ascomycota, Basidiomycota, and Chytridiomycota are the dominant fungi phyla (52, 53). At the genus level, higher abundances of *Aspergillus* and *Penicillium* were found, possibly owing to the decomposition of leaf litter and dead grass (54). Every autumn, fallen leaves and dead grass enter Hulun Lake along with the river water, causing eutrophication.

Microbial communities exhibit a close connection with their surroundings (55). Existing evidence suggests that temperature, pH, COD, TN, and TP can influence the community structure of bacteria and fungi; however, findings on this subject vary (56, 57). In Changjiang River, TP and TN affect the fungal community (58). Yellow River Lake’s fungal communities are best predicted by altitude, annual temperature, C/N ratio, dissolved organic carbon, and TN (59). In our study, pH, temperature, and DO all contributed to the community composition in Hulun Lake. This differs from the results obtained for Taihu and Poyang Lakes (18, 60). Additionally, the microbial communities were also affected by some environmental factors, but these factors did not explain all the variations.

Environmental factors may account for a fraction of microbial composition variation in the presence of diffusion and ecological drift (61). In this study, we employed VPA analysis and discovered that environmental and geographic factors accounted for <20% of the variation. Similar results have been reported for the Yellow River Delta (30). Environmental factors were more important for describing variation in the bacterial community (14.8%) than in the fungal community (12.4%). This may be because fungi have greater environmental adaptability (62, 63). In this study, both bacteria and fungi exhibited stochastic processes. However, there was a difference in the importance of these stochastic processes. This disparity may explain the unexplained community differences in VPA, possibly related to stochastic processes such as microbial growth, death, and reproduction. Several hypotheses have been proposed to explain the assembly of communities, including the “size-plasticity” and “size-dispersal” theories (64). The “size-plasticity” theory suggests that smaller organisms are more susceptible to dispersal constraints owing to their greater metabolic power and ability to survive (65). Conversely, the “size-dispersal” theory suggests that smaller organisms are more likely to disperse, making them less susceptible to dispersal limitations (66). However, neither hypothesis considers the effects of other stochastic processes, such as drift. Our findings indicate that in water, bacteria are more susceptible to diffusion-limited control than fungi. This is consistent with the “size-plasticity” hypothesis (62). In contrast, in sediments, fungi have greater dispersal limitations than bacteria, supporting the “size-dispersal” hypothesis (67). In addition to organism type, habitat may also play a role in dispersal limitation. We hypothesize that this may be due to the water being highly mobile, facilitating the dispersal of both bacteria and fungi when their viability plays a major role. In contrast, the sediment environment is relatively stable, and smaller bacteria are more conducive to dispersal. Therefore, there are seasonal differences in fungi in sediments.

Our study had some limitations. First, while amplicon sequencing revealed the diversity of uncultured microbial taxa, it differed in the precision and resolution of bacteria and fungi (68). The taxonomic sequence information generated by amplicon sequencing was also underannotated, which is particularly important for results regarding fungi (69). Second, although we looked at common environmental factors, we did not consider novel pollutants, such as microplastics and historical environmental conditions, which may also be important factors affecting microbial communities (70). Third, amplicons were used for broad studies of microbial taxa, and the functional taxa of the microorganisms were not sufficiently explored. Therefore, future research should fully investigate the diversity, function, activity, and influencing factors of microorganisms using multi-omics techniques.

### Conclusion

We investigated the bacterial and fungal communities of Hulun Lake through the simultaneous application of amplicon sequencing, CCA, and community assembly. We found that environmental factors in Hulun Lake were spatio-temporally highly variable, resulting in significant variation in bacterial and fungal communities. The differences in bacteria and fungi observed between different habitat types (water and sediments) were greater than the variation across different seasons. We found that pH, temperature, and DO were crucial environmental factors affecting communities although the combination of environmental and geographic factors accounted for <20% of the variation. In addition, we found that limited dispersal during stochastic processes played a significant role in community formation. However, it is necessary to analyze larger data sets to gain a better understanding of how these microorganisms assemble themselves. These results shed new light on the relationship between microbial communities and their environment and the ecological processes that facilitate community formation. Thus, our study establishes foundational information and contributes toward the comprehensive understanding of lake ecosystems, enhancing our understanding of lake microbial ecology.

## Data Availability

The sequence data associated with this article have been submitted to the NCBI SRA database under BioProject accession numbers PRJNA781770, PRJNA859994, PRJNA871398, PRJNA613767, and PRJNA713409.

## SUPPLEMENTAL MATERIAL

Supplemental material spectrum.03245-23-s0001.doc Tables S1, S3, S5, and S6; Fig. S1 to S4.

Table S2 spectrum.03245-23-s0002.xls Representative bacterial OTUs and the proportion of unknown sequences at different taxonomy.

Table S4 spectrum.03245-23-s0003.xls Representative fungal OTUs and the proportion of unknown sequences at different taxonomy.

ASM does not own the copyrights to Supplemental Material that may be linked to, or accessed through, an article. The authors have granted ASM a non-exclusive, world-wide license to publish the Supplemental Material files. Please contact the corresponding author directly for reuse.
