# Supplementary material for: Bacterial and fungal community structures in Hulun Lake are regulated by both stochastic processes and environmental factors
Source: Microbiol Spectr. 2024 Apr 11;12(5):e03245-23. doi: 10.1128/spectrum.03245-23 (PMC11064641; doi:10.1128/spectrum.03245-23)
Supplement: Supplemental material — Tables S1, S3, S5, and S6; Fig. S1 to S4. [file spectrum.03245-23-s0001.doc]

***Supplementary Material***

| Sample code | Description of samples in summer | | | Environmental parameters | | | | | | | |
| --- | --- | --- | --- | --- | --- | --- | --- | --- | --- | --- | --- |
|  | Name (bacteria \ fungi) | GPS | Sample Type | Temp (℃) | pH | DO (mg/L) | EC(us/cm) | NH_4_^+^-H(mg/L) | TP(mg/L) | TN(mg/L) | COD(mg/L) |
| 1 | HL1 (NS1) \ TH1 (TNS1) | N49.3095°, E117.68963° | Water & Sediment | 23.0 | 9.0 | 7.60 | 1584 | 0.20 | 0.27 | 1.8 | 62 |
| 2 | HL2 \ TH2 | N49.2519°, E117.61475° | Water | 23.0 | 9.0 | 7.38 | 1589 | 0.72 | 0.28 | 3.0 | 61 |
| 3 | HL3 \ TH3 | N49.22355°, E117.49542° | Water | 23.0 | 9.0 | 7.34 | 1582 | 0.02 | 0.23 | 1.2 | 50 |
| 4 | HL4 (NS4) \ TH4 (TNS4) | N49.1615°, E117.41966° | Water & Sediment | 23.0 | 8.9 | 7.56 | 1606 | 0.46 | 0.22 | 1.0 | 57 |
| 5 | HL5 (NS5) \ TH5 (TNS5) | N48.92639°, E117.13842° | Water & Sediment | 23.5 | 9.0 | 7.56 | 1697 | 3.00 | 0.30 | 3.7 | 57 |
| 6 | HL6 (NS6) \ TH6 (TNS6) | N48.85732°, E117.01166° | Water & Sediment | 24.0 | 9.0 | 7.87 | 1721 | 11.30 | 0.22 | 1.6 | 44 |
| 7 | HL7 (NS7) \ TH7 (TNS7) | N48.77977°, E117.05325° | Water & Sediment | 24.0 | 9.1 | 8.19 | 1731 | 0.02 | 0.20 | 1.2 | 59 |
| 9 | HL9 (NS9) \ TH9 (TNS9) | N48.71831°, E117.30044° | Water & Sediment | 24.0 | 9.1 | 7.61 | 1736 | 0.02 | 0.19 | 2.0 | 70 |
| 10 | HL10 (NS10) \ TH10 (TNS10) | N48.95555°, E117.47722° | Water & Sediment | 24.0 | 9.1 | 7.74 | 1732 | 45.00 | 0.19 | 2.0 | 63 |
| 11 | HL11 (NS11) \ TH11 (TNS11) | N48.95611°, E117.73694° | Water & Sediment | 24.0 | 9.0 | 7.64 | 1741 | 1.31 | 0.18 | 3.4 | 54 |
| 12 | HL12 (NS12) \ TH12 (TNS12) | N48.96851°, E117.63537° | Water & Sediment | 24.0 | 9.1 | 7.60 | 1740 | 0.15 | 0.22 | 1.7 | 59 |
| 13 | HL13 \ TH13 | N48.20388°, E117.86111° | Water | 24.0 | 9.1 | 7.68 | 1707 | 0.76 | 0.24 | 2.6 | 64 |
| 14 | HL14 (NS14) \ TH14 (TNS14) | N49.14039°, E117.7512° | Water & Sediment | 25.8 | 8.8 | 8.18 | 1048 | 0.10 | 0.27 | 0.9 | 54 |
| 15 | HL15 \ TH15 | N48.85655°, E117.22237° | Water | 24.0 | 9.0 | 7.68 | 1710 | 0.93 | 0.29 | 1.7 | 58 |
| 16 | HL16 (NS16) \ TH16 (TNS16) | N49.00232°, E117.44392° | Water & Sediment | 25.0 | 9.0 | 8.43 | 1718 | 1.23 | 0.30 | 3.3 | 59 |
| 17 | HL17 (NS17) \ TH17 (TNS17) | N49.07006°, E117.5942° | Water & Sediment | 25.0 | 9.0 | 8.09 | 1738 | 0.02 | 0.29 | 3.9 | 55 |
| 18 | HL18 (NS18) \ TH18 (TNS18) | N49.30447°, E118.04613° | Water & Sediment | 25.5 | 8.4 | 8.20 | 168 | 0.19 | 0.10 | 1.4 | 19 |
| 19 | HL19 (NS19) \ TH19 (TNS19) | N49.42896°, E117.78493° | Water & Sediment | 26.3 | 8.3 | 7.52 | 238 | 0.02 | 0.09 | 1.3 | 20 |
| 20 | HL20 (NS20) \ TH20 (TNS20) | N48.75912°, E117.04425° | Water & Sediment | 28.0 | 8.3 | 7.08 | 336 | 0.02 | 0.26 | 2.0 | 13 |
| 21 | HL21 \ TH21 | N48.34636°, E117.48103° | Water | 26.5 | 8.9 | 10.70 | 1461 | 0.02 | 0.18 | 3.8 | 80 |
| 22 | HL22 \ TH22 | N47.96489°, E117.71295° | Water | 26.0 | 8.5 | 7.44 | 354 | 0.02 | 0.05 | 0.6 | 44 |
| 23 | HL23 (NS23) \ TH23 (TNS23) | N48.96077°, E117.74629° | Water & Sediment | 29.5 | 8.8 | 7.18 | 446 | 0.02 | 0.05 | 0.6 | 31 |

**Tables and figures**

**Table S1**. Description of samples point and environmental parameters of Hulun Lake investigated in this study.

| Sample code | Description of samples in winter | | | Environmental parameters | | | | | | | |
| --- | --- | --- | --- | --- | --- | --- | --- | --- | --- | --- | --- |
|  | Name (bacteria \ fungi) | GPS | Sample Type | Temp (℃) | pH | DO (mg/L) | EC(us/cm) | NH_4_^+^-H(mg/L) | TP(mg/L) | TN(mg/L) | COD(mg/L) |
| 1 | WHL1 (WHLN1) \ TWHL1 (TWHLN1) | N49.26935°, E117.61502° | Water & Sediment | 0.8 | 8.9 | 14.7 | 1773 | 0.14 | 0.11 | 1.57 | 233 |
| 2 | WHL2 (WHLN2) \ TWHL2 (TWHLN2) | N49.11396°, E117.41777° | Water & Sediment | 0.4 | 9.0 | 14.9 | 1927 | 0.05 | 0.15 | 1.76 | 140 |
| 3 | WHL3 (WHLN3) \ TWHL3 (TWHLN3) | N48.98265°, E117.21447° | Water & Sediment | 2.4 | 8.8 | 14.2 | 1941 | 0.08 | 0.19 | 1.95 | 176 |
| 4 | WHL4 (WHLN4) \ TWHL4 (TWHLN4) | N48.76506°, E117.04871° | Water & Sediment | -0.3 | 8.8 | 12.7 | 1672 | 0.14 | 0.04 | 1.52 | 121 |
| 5 | WHL5 (WHLN5) \ TWHL5 (TWHLN5) | N48.76636°, E117.14759° | Water & Sediment | -0.1 | 8.9 | 15.6 | 1957 | 0.14 | 0.11 | 1.79 | 136 |
| 6 | WHL6 (WHLN6) \ TWHL6 (TWHLN6) | N49.00598°, E117.68552° | Water & Sediment | -1.2 | 9.0 | 15.5 | 1810 | 0.09 | 0.12 | 4.16 | 233 |
| 7 | WHL7 (WHLN7) \ TWHL7 (TWHLN7) | N49.13807°, E117.67792° | Water & Sediment | -1.3 | 8.9 | 14.6 | 1844 | 0.16 | 0.11 | 1.64 | 142 |
| 8 | WHL8 (WHLN8) \ TWHL8 (TWHLN8) | N49.00801°, E117.59022° | Water & Sediment | -0.3 | 8.8 | 15.5 | 1932 | 0.1 | 0.13 | 2.41 | 216 |
| 9 | WHL9 (WHLN9) \ TWHL9 (TWHLN9) | N48.75205°, E117.47722° | Water & Sediment | -0.4 | 8.9 | 15.4 | 1883 | 0.06 | 0.83 | 6.43 | 218 |
| 10 | WHL10 (WHLN10) \ TWHL10 (TWHLN10) | N48.81107°, E117.14759° | Water & Sediment | 3.3 | 8.5 | 15.3 | 1937 | 0.21 | 0.14 | 1.82 | 229 |
| 18 | WHL18 \ TWHL18 | N49.3042°, E118.3042° | Water | -0.2 | 7.7 | 15.1 | 30 | 0.14 | 0.02 | 0.95 | 18 |
| 19 | WHL19 \ TWHL19 | N48.48648°, E117.75479° | Water | -0.3 | 8.1 | 17.2 | 155 | 0.15 | 0.01 | 0.88 | 20 |
| 20 | WHL20 (WHLN11) \ TWHL20 (TWHLN11) | N48.75888°, E117.04403° | Water & Sediment | 0.4 | 7.9 | 14.37 | 748 | 0.06 | 0.06 | 1.21 | 282 |
| 21 | WHL21 \ TWHL21 | N48.34636°, E117.48103° | Water | -0.2 | 7.1 | 4.7 | 4700 | 0.31 | 0.3 | 6.12 | 295 |
| 22 | WHL22 \ TWHL22 | N47.96475°, E117.71347° | Water | -0.2 | 8.4 | 19.19 | 232 | 0.09 | 0.01 | 1.39 | 50 |
| 23 | WHL23 \ TWHL23 | N48.96455°, E117.72968° | Water | -0.7 | 9.0 | 15 | 108 | 0.14 | 0.03 | 1.53 | 224 |
| 24 | WHL24 \ TWHL24 | N49.26488°, E119.76117° | Water | 0 | 8.3 | 11.1 | 235 | 0.13 | 0.01 | 0.29 | 13 |
| 25 | WHL25 \ TWHL25 | N49.26101°, E119.72915° | Water | 0 | 7.4 | 9.8 | 253 | 0.14 | 0.19 | 0.58 | 16 |
| 26 | WHL26 \ TWHL26 | N49.21795°, E118.90552° | Water | -0.1 | 8.0 | 11.5 | 210 | 0.08 | 0.03 | 1.03 | 17 |
| 27 | WHL27 \ TWHL27 | N49.27916°, E119.27917° | Water | -0.1 | 7.9 | 9.2 | 2560 | / | / | / | / |
| 28 | WHL28 \ TWHL28 | N48.40848°, E117.58324° | Water | -0.3 | 7.7 | 4.7 | 1548 | 0.09 | 0.01 | 1.31 | 83 |

Note: Temp, temperature; DO, dissolved oxygen; EC, conductivity; NH_4_^+^-N, ammonia nitrogen; TP, total phosphorus; TN, total nitrogen; COD, chemical oxygen demand.

**Table S3 |** Bacterial and fungal α diversity index in different groups.

| Microbial type | group | sobs | goods_coverage | Richness estimator | | Diversity index | |
| --- | --- | --- | --- | --- | --- | --- | --- |
|  |  |  |  | ace | Chao 1 | shannon | simpson |
| Bacterial | HL | 1913.32±425.97 | 1.00±0.00 | 2541.85±735.51 | 2552.46±695.23 | 5.81±1.19 | 0.90±0.09 |
|  | NS | 3556.69±555.64 | 0.99±0.00 | 4311.60±548.93 | 4425.47±581.51 | 8.68±0.98 | 0.99±0.01 |
|  | WHL | 2423.43±758.30 | 1.00±0.00 | 3342.73±708.84 | 3351.34±732.06 | 5.91±0.93 | 0.93±0.06 |
|  | WHLN | 4288.82±1123.95 | 1.00±0.00 | 4954.58±1074.06 | 5099.92±1091.16 | 7.62±1.26 | 0.97±0.03 |
| Fungal | TH | 56.18±17.16 | 0.94±0.03 | 95.04±34.13 | 91.97±36.19 | 4.15±0.93 | 0.86±0.11 |
|  | TNS | 50.00±14.77 | 0.95±0.02 | 71.44±26.89 | 72.39±34.52 | 3.92±0.99 | 0.83±0.16 |
|  | TWHL | 70.43±34.41 | 0.92±0.04 | 110.21±56.16 | 100.84±51.89 | 4.41±1.67 | 0.84±0.21 |
|  | TWHLN | 71.91±22.44 | 0.93±0.04 | 105.12±48.47 | 100.42±39.79 | 4.85±0.67 | 0.92±0.04 |

**Table S5**. Correlation between the environmental factors and the β - NTI matrix by the Mantel test of bacterial and fungal communities.

| environmental factors | Bacteria | | | | Fungi | | | |
| --- | --- | --- | --- | --- | --- | --- | --- | --- |
|  | H-WH | | N-WN | | TH-TWH | | TN-TWN | |
|  | r | P | r | P | r | P | r | P |
| Temp | 0.03267 | 0.165 | -0.05985 | 0.773 | 0.01284 | 0.372 | 0.05259 | 0.139 |
| pH | -0.02576 | 0.742 | -0.06176 | 0.932 | -0.006598 | 0.55 | -0.03493 | 0.685 |
| DO | -0.003428 | 0.556 | -0.04032 | 0.63 | 0.04418 | 0.137 | 0.0691 | **0.043^*^** |
| EC | -0.03306 | 0.816 | -0.02802 | 0.579 | 0.01701 | 0.338 | -0.02435 | 0.474 |
| NH_4_^+^-N | -0.02872 | 0.771 | -0.02154 | 0.675 | -0.0451 | 0.838 | 0.07424 | **0.024^*^** |
| P | -0.05214 | 0.917 | 0.002129 | 0.427 | 0.007371 | 0.444 | -0.01667 | 0.556 |
| N | -0.02664 | 0.76 | -0.05289 | 0.796 | -0.04449 | 0.833 | 0.0255 | 0.316 |
| COD | -0.01916 | 0.714 | -0.05052 | 0.772 | 0.008953 | 0.426 | 0.0467 | 0.205 |

Note： * P＜0.05

**Table S6**. Topological properties of the microbial phylogenetic molecular networks of bacterial and fungal.

| Network metrics | ITS | | 16S | |
| --- | --- | --- | --- | --- |
|  | TH-TWH | TN-TWN | H-WH | N-WN |
| Nodes | 82 | 84 | 99 | 87 |
| Edge | 283 | 213 | 1329 | 465 |
| Average Degree^a^ | 6.902 | 5.071 | 26.848 | 10.69 |
| Network Diameter | 5 | 7 | 5 | 7 |
| Graph Density | 0.085 | 0.061 | 0.274 | 0.124 |
| Modularity^b^ | 0.43 | 0.644 | 0.387 | 0.4 |
| Average Clustering Coefficient^c^ | 0.228 | 0.233 | 0.353 | 0.256 |
| Average Path Length^d^ | 1.828 | 2.191 | 1.927 | 2.347 |

a Average degree refers to the average connectivity for nodes in the whole network. Higher average degree means a more complex network.

b Modularity measures how well a network is able to be separated into modules.

c Average clustering coefficient is the average clustering coefficient of nodes in the whole network. Higher Average clustering coefficient means more tightened network structure.

d Average path length is similar to average geodesic distance. A smaller average path length all the nodes in the network are closer.


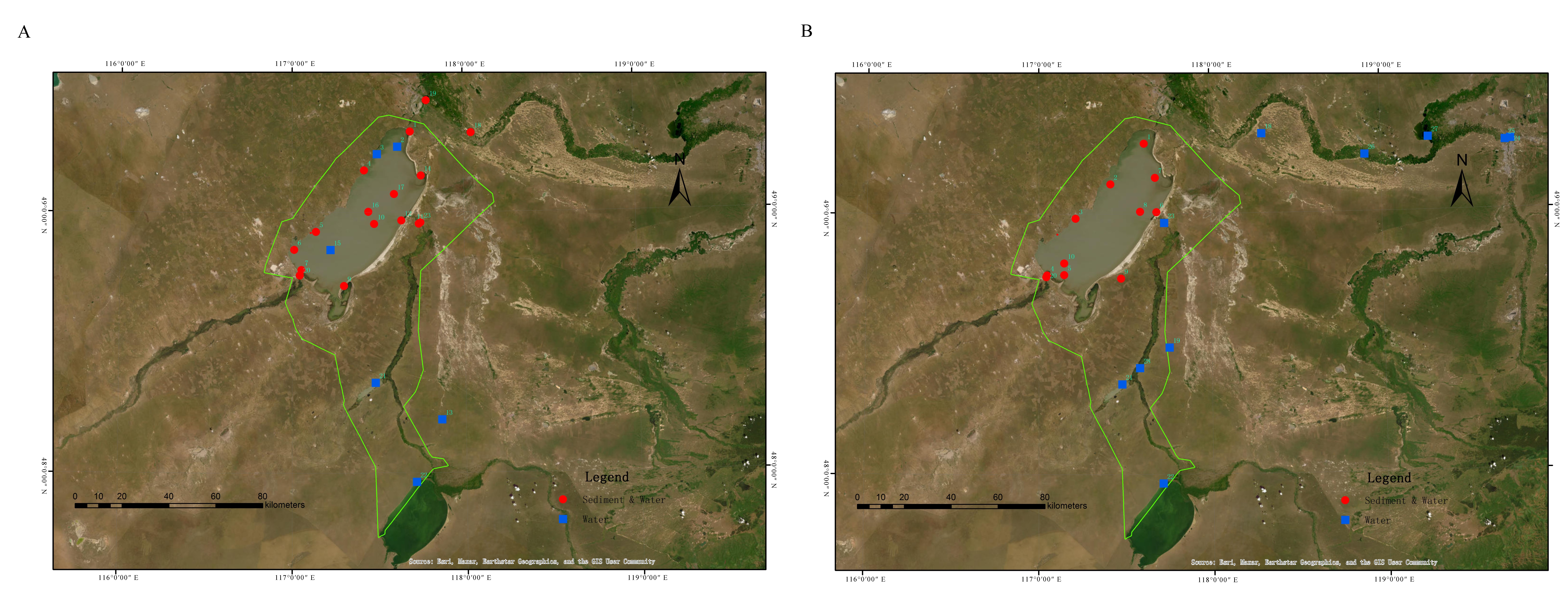


**Fig. S1**. Distribution map of (A) the summer and (B) the winter sampling points in the Hulun Lake Reserve. The latitude and longitude of the sampling points are shown in Table S1.

**
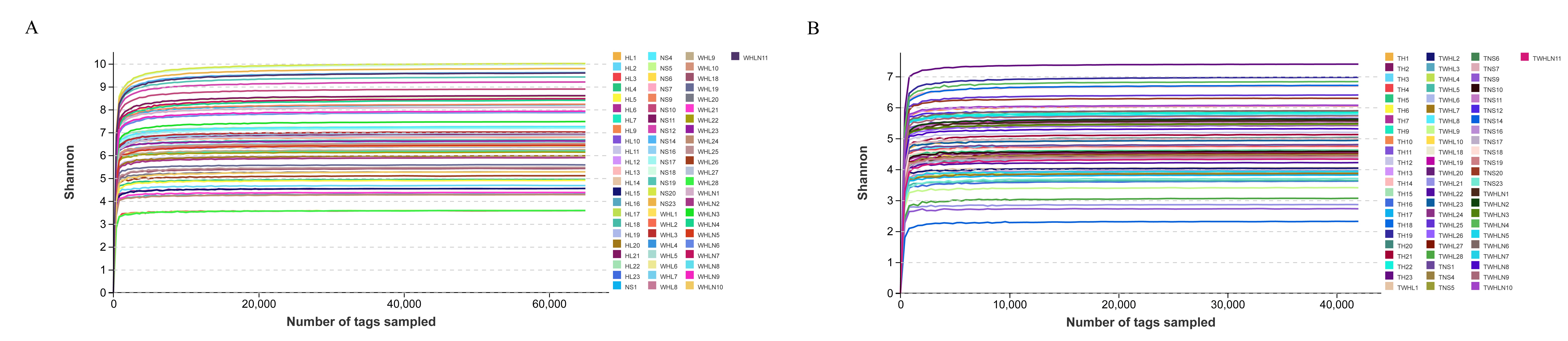
**

**Fig. S2** Rarefaction curves of the Shannon index across (A)bacterial communities and (B) fungal communities from Hulun Lake.


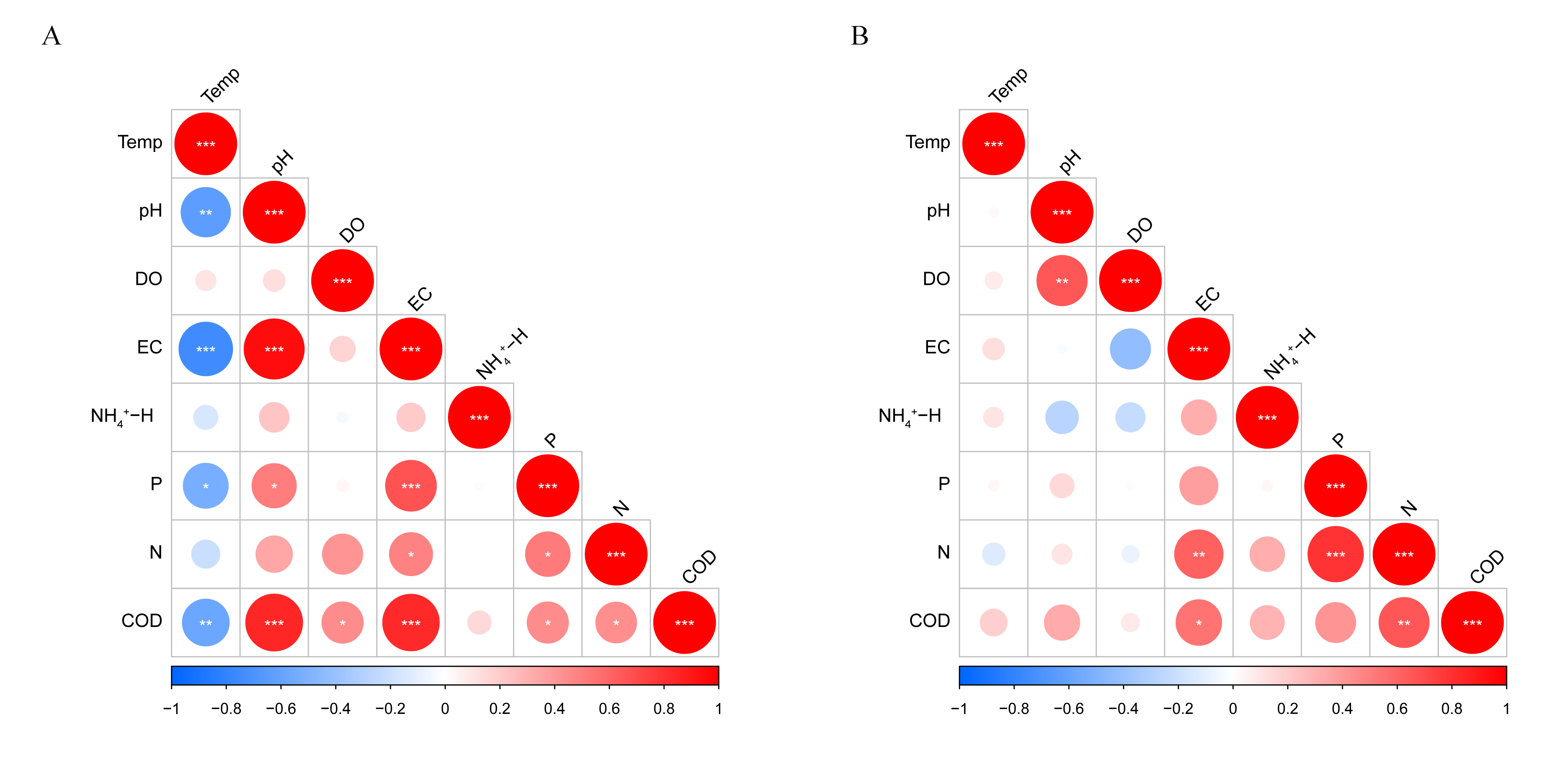


**Fig. S3** The correlation analysis between environmental factors of (A) summer and (B) winter. Environmental factors with significant correlations are represented by * (*: P＜0.05, **: P＜0.01, ***: P＜0.001). Blue indicate negative correlations and red indicate positive correlations. The circle size and color represent the degree of correlation.


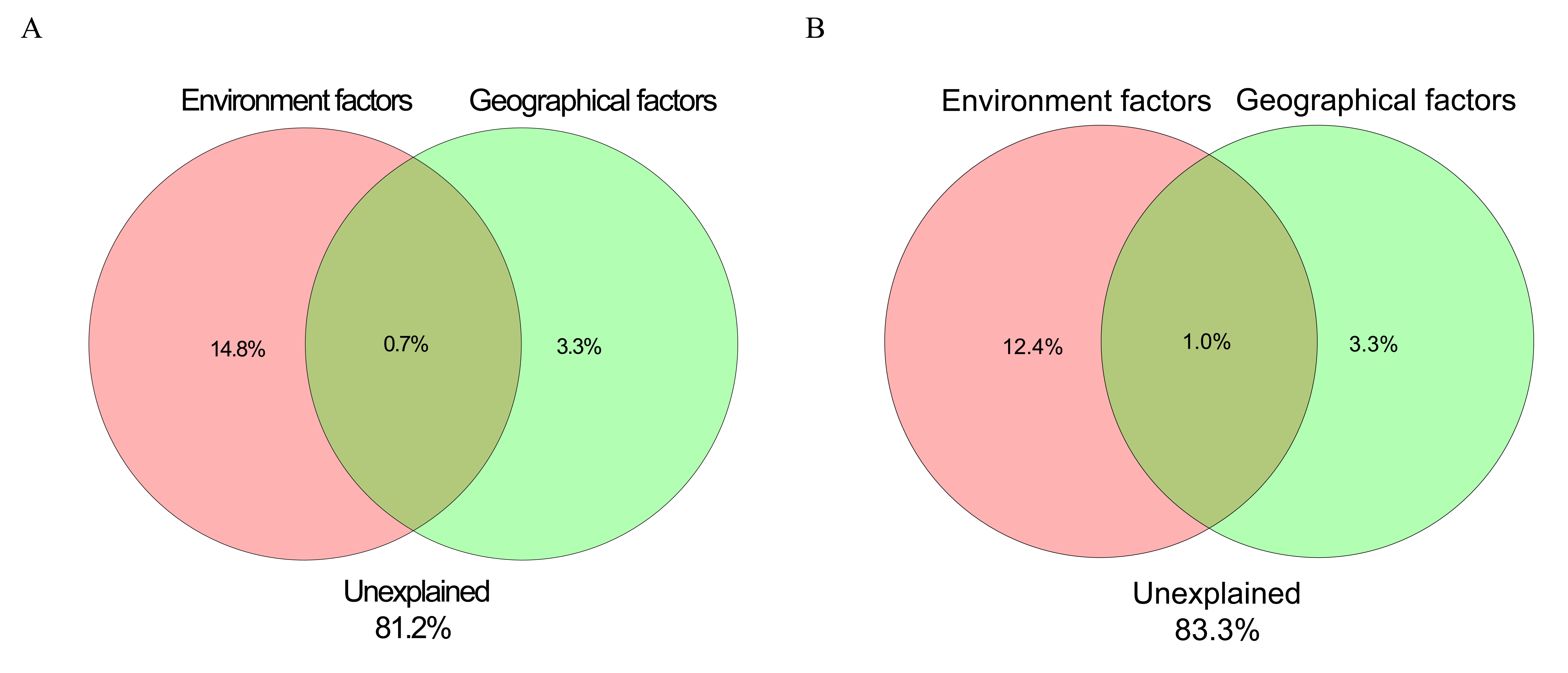


**Fig. S4** Variation partition analysis (VPA) showing the contribution of environmental factors and Geographical factors for community variance of (C) bacteria and (D) fungi.
